# Regulation of gene expression by microRNA in HCV infection and HCV–mediated hepatocellular carcinoma

**DOI:** 10.1186/1743-422X-11-64

**Published:** 2014-04-01

**Authors:** Priyanka Gupta, Murray J Cairns, Nitin K Saksena

**Affiliations:** 1 Retroviral Genetics Division, Centre for Virus Research, Westmead Millennium Institute, University of Sydney, Darcy Road, Sydney, Westmead NSW 2145, Australia; 2 School of Biomedical Sciences and Pharmacy, Faculty of Health and the Hunter Medical Research Institute, The University of Newcastle, Callaghan NSW 2308, Newcastle, Australia; 3 Centre for Virus Research, Westmead Millennium Institute, Darcy Road, Sydney, Westmead NSW 2145, Australia

**Keywords:** Hepatitis C virus, Hepatocellular carcinoma, miRNA, mRNA

## Abstract

MicroRNA (miRNA) exert a profound effect on Hepatitis C virus (HCV) replication and on the manifestation of HCV-associated hepatocellular carcinoma (HCC). miR-122 in particular, is highly enriched in liver and has been shown to interact with HCV, suggesting this virus has evolved to subvert and manipulate the host gene silencing machinery in order to support its life cycle. It is therefore likely that miR-122 and other miRNAs play an important role in the pathophysiology of HCV infection. The changes in post-transcriptional gene regulation by the miRNAs may play a key role in the manifestation of chronic liver disease and hepatocellular carcinoma. Understanding of HCV-host miRNA interactions will ultimately lead to the design of therapeutic modalities against HCV infection and HCV-mediated HCC and may also provide important biomarkers that direct treatment options. Here, we review the current knowledge on the role of miRNA and gene expression on HCV infection and hepatocellular carcinoma, in addition to the possible role of miRNA as future therapeutic targets.

## Introduction

HCV infection is a prevalent disease with approximately 150 million individuals (3%) chronically infected worldwide [1]. About 20–67% of patients experience spontaneous clearance of the virus and this is an event occurring principally during the first 3 months after clinical onset of disease [2-4]. Chronic disease should be considered if viremia persists for more than 6 months. Progression of liver disease occurs over 20–30 years and is accelerated in the presence of cofactors such as alcohol consumption, diabetes mellitus, older age of acquisition, human immunodeficiency virus (HIV) co-infection, or co-infection with other hepatotropic viruses [5]. Depending on the presence of co-factors, between 10% and 40% of patients with chronic HCV infection will develop cirrhosis [6]. Approximately 4% per year may suffer death related to the complications of cirrhosis, whereas HCC occurs in this population at an estimated incidence of 1–5% per year [7]. In the absence of a protective vaccine against HCV infection, Interferon (IFN)/Ribavirin therapy is currently the mainstay in the management of HCV infection. Because this therapy is poorly tolerated and is effective only in a subset of HCV infections [8-11], there is a need of interferon-free therapies or direct acting antiviral drugs e.g. Telaprevir that inhibit the HCV NS3/4A protease and achieve sustained viral clearance in combination with the standard therapy.

miRNAs are small (17–23 nucleotide) non-coding RNAs that serve as post-transcriptional regulators of gene expression, many of which are conserved across metazoans [12]. The first miRNA, lin-4, was discovered by Lee *et al.* in 1993 in C. *elegans*, where it was found to negatively regulate protein levels of LIN-14 [13]. miRNAs are great candidates as regulators of host and viral gene expression because of their small size, flexibility in function. The functions of miRNA are diverse ranging from development control of central physiological processes such as apoptosis, proliferation and differentiation to involvement in disease e.g. cancer [14,15]. It has been estimated that miRNAs regulate more than 60% of human protein coding genes using *in silico* approaches and over 2,000 human mature miRNAs have been annotated (miRBase v19.0; http://www.mirbase.org/) [16].

### Biogenesis of miRNA

**Figure 1:**
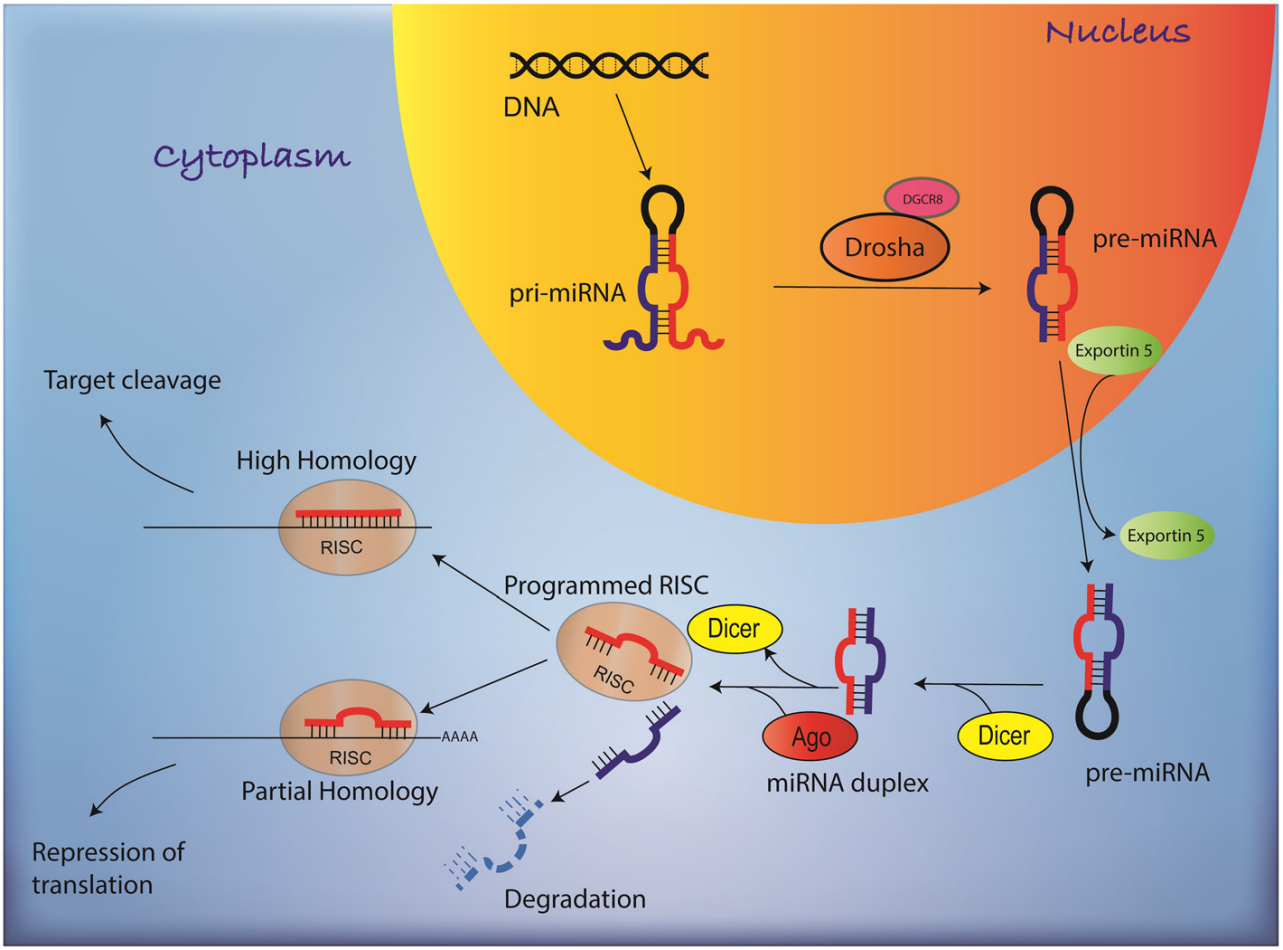
**Biogenesis of miRNA.** miRNAs are transcribed from the genes by RNA polymerase II. The microprocessor complex (Drosha and DGCR8) processes the primary miRNA transcripts into pre-miRNA. The pre-miRNA is then exported into the cytoplasm by exportin-5 and processed into a RNA duplex by ribonuclease Dicer in association with TRBP. The end product is 22 bp double stranded RNA. One strand of the dsRNA remains bound to the dicer to form the mature miRNA while the other RNA strand is generally degraded. The remaining strand is then assembled into the RISC with Ago 2 and can target gene silencing through either cleavage or translational repression.

Most miRNAs are transcribed from intergenic regions, introns and exons by RNA polymerase II. The initial RNA transcript is a RNA precursor called a primary miRNA (pri-miRNA) [17-20] (Figure 1). pri-miRNA ranges from 200 nucleotides to several thousand nucleotides in length and is known to form highly structured stem loop [21,22]. The cellular RNase III enzyme ‘Drosha’ cleaves this stem loop with the help of cofactor DiGeorge syndrome critical region gene 8 (DGCR8) in vertebrates and ‘Pasha’ in invertebrates [23-25] (Figure 1). The cleavage produces an RNA hairpin intermediate around 70 nucleotides, known as the precursor-miRNA or pre-miRNA with a characteristic two nucleotide 3′ overhang [23]. The next step in the biogenesis of miRNA is the nuclear export of the pre-miRNA hairpin by a heterodimer consisting of exportin 5 and the GTP bound form of cofactor Ras-related nuclear protein (RAN), which recognize and bind the two- nt 3′ overhang of pre-miRNA [26,27] (Figure 1). In the cytoplasm, another cellular RNase III enzyme called Dicer binds to the structured DNA with cofactor Transactivation response RNA binding protein (TRBP) to perform a second cleavage. The end product is a two nt 3′ overhang approximately 17–22 bp double stranded RNA. One strand of the dsRNA remains bound to the Dicer to form the mature miRNA while the other RNA strand is generally degraded. The remaining strand is then integrated into a protein complex called the RNA-induced silencing complex (RISC) with the help of dicer [28,29]. Argonaute-2 (Ago-2) protein is a catalytically active ribonucleoprotein and is a key component in RISC [30,31]. Mature miRNA bound to the active RISC binds to the target sites at 3′ UTR of mRNA leading to direct inhibition of translation or mRNA target degradation by Ago2 protein **(**Figure 1) [30]. Vertebrates only need partial complementary miRNA to recognize their targets, however, it is critical to have a high degrees of complementary base pairing of miRNA nucleotides through the nucleotides 2–8 referred to as the “seed sequence” [32,33].

### Evidence suggests that miRNAs play a significant role in HCV replication

Several miRNAs have been identified in various studies to play a key role in regulating the virus replication and pathogenesis during HCV infection [34-39]. miR-122 is the most abundant liver-specific miRNA and has been demonstrated by several studies to be required for HCV replication in infected cells [34,40,41]. Sequestration of miR-122 in liver cell lines strongly reduced HCV translation, whereas addition of miR-122 stimulated HCV translation in liver cell lines [42]. Study on liver biopsies of chronic hepatitis patients demonstrated that miR-122 level in primary non-responding subjects was lower than in early virological responding subjects, regardless of the viral genotype [43].

miR-122 is the miRNA that exerts a positive effect on viral replication in cell culture by binding to the viral 5′ Untranslated region (UTR), as shown by *in vitro* studies [34,44]. miR-122 was shown to form an oligomeric complex in which one miR-122 molecule binds to the 5′ UTR of HCV RNA with 3′ overhanging nucleotides, masking the 5′ terminal sequences of HCV genome [45]**(**Table 1). This also suggested that these 5′ terminal viral sequences are thus protected from nucleolytic degradation [45]. The specific internal nucleotides and the 3′ terminal nucleotides in miR-122 were shown to be absolutely required for maintaining HCV RNA abundance rather than influencing their function [45]. It was shown that overexpression of miR-122 significantly suppressed the interferon-stimulated response element (ISRE), that functions as an enhancer to promote the induction of transcription by alpha/beta interferons [46-51] (Table 1). Contrary to that, suppression of miR-122 function enhanced the ISRE activity, by decreasing expression of suppressor of cytokine signaling 3 (SOCS3) [51] (Table 1). The decrease in SOCS3 level was not mediated by the target gene expression, but by enhanced methylation at SOCS3 gene promoter [51]. The authors also suggested that the data is supportive of use of a combination therapy of IFN and silencing of miR-122 function (i.e. antisense oligonucleotides of miR-122) as a therapeutic option in near future [51]. Interestingly, Ago proteins were shown to be required for miR-122 to activate translation via the HCV 5′-UTR [52,53]. Cyclin G1 was shown to be a target for miR-122 and use of miR-122 inhibitor was reported to prevent the alcohol-induced increase in HCV RNA and protein levels [54] (Table 1). miR-122 was also shown to protect HCV RNA from 5′decay by targeting 5′ exonuclease Xrn1 [55] (Table 1). Moreover, exogenous expression of miR-122 supports efficient HCV RNA replication [56] and/or effective virus propogation in non- permissive cell line [57,58]. miR-122 has been shown to be also involved in cell cycle progression in hepatoma cell line [59]. miR-141 mediated suppression of Deleted in Liver Cancer (DLC-1) (a Rho GTPase-activating protein) enhances viral replication in HCV-infected primary human hepatocytes [35] (Table 1). HCV replication was shown to be dependent on the miR-141 induction and antagomir-mediated knockdown of miR-141 severely inhibited the replication of HCV [35]. Interestingly, artificially increasing intracellular miR-141 induced virus replication in the infected hepatocytes, thereby confirming its functional importance [35]. miR- 130a expression is up-regulated in liver biopsy from HCV-infected patients and facilitated HCV replication by targeting antiviral Interferon inducible trans-membrane protein (IFITM); knockdown of miR-130a inhibited HCV replication in hepatocytes [36] (Table 1). miR- 130a expression is up-regulated in liver biopsy from HCV infected patients and similar observation was also reported on miR-130a- mediated regulation of viral replication in HCV-infected cells [60]. Contrary to the above studies, a recent study by Li *et al.* demonstrated that miR-130a inhibited HCV replication by restoring the innate immune response [61] (Table 1). miR-21 was shown to target myeloid differentiation factor 88 (MyD88) and interleukin-1 receptor-associated kinase 1 (IRAK1), which are involved in type I IFN production induced by the HCV [62]. This subsequently leads to repressed IFN mediated antiviral response, thereby promoting viral replication [62] (Table 1).

**Table 1:** Selected miRNA and their cognate gene targets relevant to HCV infection

	**miRNA**	**Target**	**Function**
**miRNAs that promote HCV replication**	miR-122	HCV 5'-UTR	Promote HCV replication [34,40,41] and IRES mediated HCV translation [52]
		SOCS3	Enhance methylation at SOCS3 gene promoter, inhibits IFN-induced ISRE activity [51]
		Cyclin G1	Promote viral replication induced by alcohol [54]
		Xrn1	Inhibit 5′decay of HCV RNA [55]
	miR-141	DLC	Up-regulated in HCV infection [35]
	miR-130a	IFITM	Inhibits type I IFN signaling and promote HCV replication [36]
	miR-21	MyD88 and IRAK1	Negatively regulate IFN signaling [62]
**miRNAs that inhibit HCV replication**	miR-196	NS5A region in HCV genome	Inhibition of HCV replication [37]
		BACH1	Inhibits HCV RNA and NS5A protein expression [63]
	miR-448	Core region of HCV genome	Inhibition of HCV replication [37]
	let-7b	NS5B and 5'UTR regions in HCV genome	Reduces HCV infectivity [38]
		BACH1	Repression of HCV expression [64]
	miR-199a	HCV 5'-UTR	Inhibits viral replication [39]
	miR-130a	IFNα/IFN β ?	Inhibits HCV RNA replication by increased expression of IFN-α/IFN- β and the ISGs [61]

*Abbreviations*: *UTR* Un-translated region, *SOCS3* suppressor of cytokine signaling 3, *ISRE* interferon stimulated responsive element, *Xrn1* exoribonuclease enzyme, *DLC-1* deleted in liver cancer 1, *IFITM* interferon inducible transmembrane protein, *IRAK1* interleukin-1 receptor-associated kinase 1, *MyD88* myeloid differentiation factor 88, *BACH1* transcription regulator protein, *ISGs* interferon stimulated genes.

**Figure 2:**
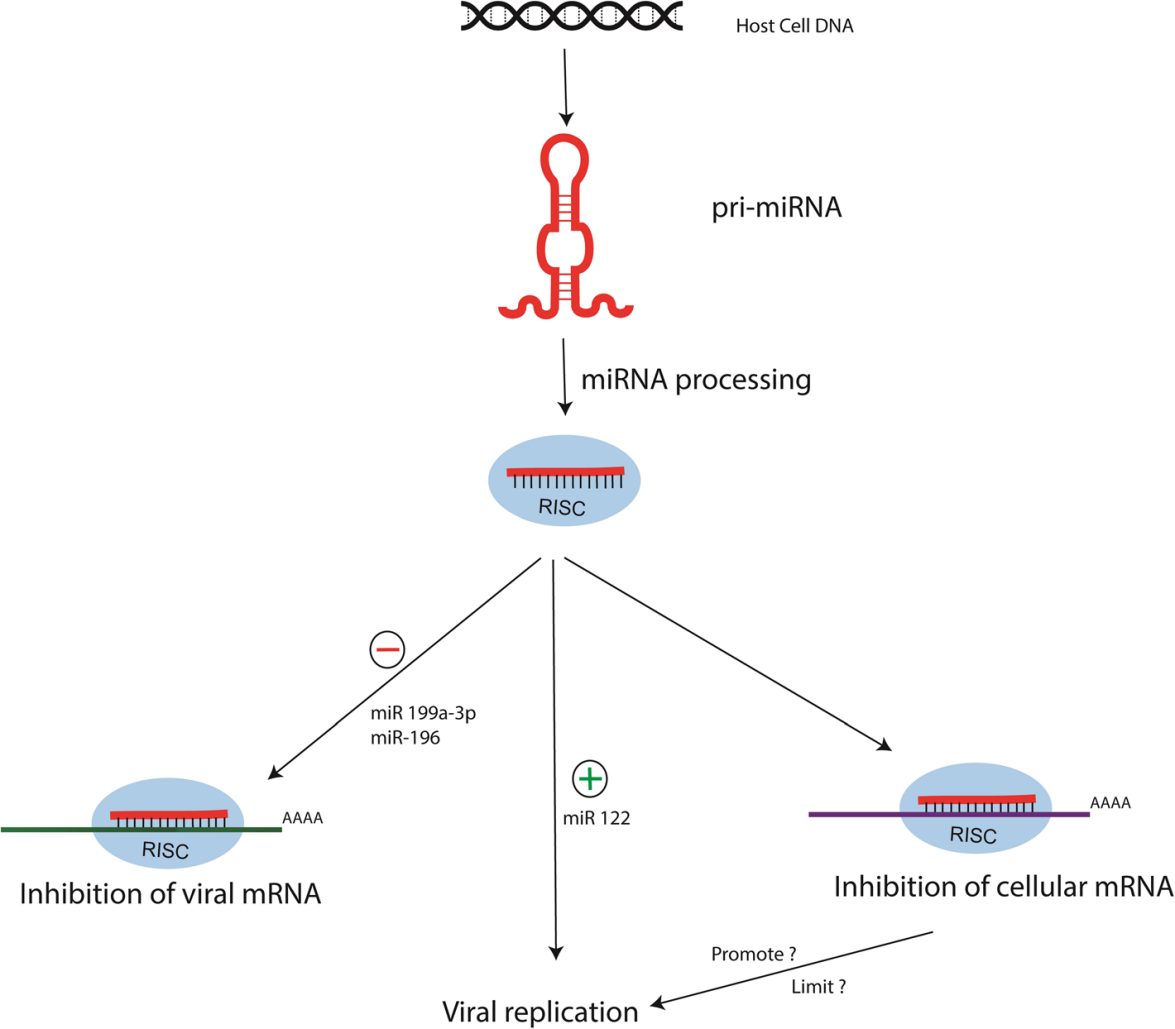
**Mechanisms by which the cellular miRNA can affect the replication of HCV.** Cellular miRNAs may bind the viral mRNAs and thereby promote the viral replication e.g. miR-122, a liver specific miRNA that can promote HCV RNA replication. The cellular miRNAs can bind to the viral genes and limit the HCV RNA replication e.g. miR-199a-3p and miR-196. Finally cellular miRNAs might promote or limit the viral replication by inhibition of the cellular mRNAs.

Finally, cellular miRNAs might indirectly promote or limit virus replication through regulation of their endogenous mRNA targets (Figure 2). In this case, viruses could benefit from reshaping the cellular miRNA environment. Cellular miRNAs may mediate the recognition of viral mRNAs (green) by RISC (blue circle) and thereby limit virus replication (Figure 2). IFN- β was shown by the researchers to modulate host miRNAs, five of which showed anti-HCV effects in a viral sequence specific manner [37]. IFN-induced miRNAs miR-196 and miR-448 have been shown to directly target CORE and NS5A coding region of HCV genomic RNA for inhibition of viral replication [37] (Table 1). miR-196 has a target site in the NS5A coding region of HCV and has been shown to inhibit the viral replication by 50-80% [37] (Figure 2 and Table 1). In addition to direct targeting of HCV RNA, miR-196 has been shown to target Bach1, a repressor of the anti-oxidative and anti-inflammatory heme oxygenase 1 (HMOX1) [63] (Table 1). The addition of miR-196 mimic in functional assays significantly down-regulated Bach-1 levels with up-regulation of HM0X1, inhibiting HCV expression [63]. let-7b has been shown to elicit anti-HCV activity by targeting the NS5B and 5′-UTR of HCV genome [38]. let-7 miRNA has been shown to target Bach1 and thereby upregulate heme oxygenase gene expression and this action leads to the attenuation of the liver injury [64] (Table 1). miR-199a-3p had 80-90% inhibitory effect on HCV replication and the identification of miR-199a-3p binding sites in the 5′UTR internal ribosome entry site (IRES) of HCV RNA (genotypes 1b and 2a) suggested a direct interaction between these molecules [39] (Table 1).

### Significant role of miRNA expression in liver cancer

Specific changes in miRNA expression patterns have been shown in HCC as compared with adjacent normal liver tumor tissues, or liver cirrhosis that correlated with the disease outcome [39,65-67]. Using a human miRNA microarray, Murakami *et al.* analysed the miRNA expression profiles in 25 pairs of hepatocellular carcinoma (HCC) and adjacent non-tumorous tissue (NT) and nine additional chronic hepatitis (CH) specimens [66]. This study found out that seven mature and one precursor miRNAs exhibited significant differential expression patterns between the HCC and NT samples, with miR-18, precursor miR-18, and miR-224 up-regulated in HCC samples and miR-199a*, miR-195, miR-199a, miR-200a, and miR-125a down-regulated in HCC samples [66]. In addition, the analysis of a small number of HCC samples also compared miRNA expression in tumors differing in differentiation state. The expression levels of four miRNAs (miR-92, miR-20, miR-18 and precursor miR- 18) were significantly higher in poorly differentiated HCC samples suggesting that these miRNAs can contribute to tumorigenesis and the loss of tumor differentiation [66]. By contrast, miR-99a expression exhibited a positive correlation between expression levels and the degree of tumor differentiation suggesting that this miRNA might contribute to the differentiation of tumor [66].

The expression of 182 precursors and 196 mature miRNAs was profiled in 43 specimens of HCC, 43 adjacent benign liver tissues, and in 12 normal liver tissues using real time PCR [67]. A total of 16 miRNAs were differentially expressed in HCC tissues as compared to the adjacent non-cancerous tissues. Dys-regulation of miR-199a, miR-199a* was reported by both Murakami and Jiang, supporting their role in the development of HCC [66,67]. miR-21 and miR-221 were also shown to be up-regulated supporting their role in the hepato-carcinogenesis. Comparing the miRNA expression patterns with the survival time revealed two general categories of patients – one with predominantly lower miRNA expression and poor survival and the other with higher miRNA expression and good survival (P < 0.05) [67]. Jiang *et al.* thus characterized a panel of 19 cell cycle related miRNAs as prognostic markers of HCC because the expression of these miRNAs correlated with survival in HCC patients [67].

In another study, miRNA expression profiles of HCC and cirrhotic liver samples were analyzed by microarray [68]. 35 miRNAs were significantly dysregulated when compared to cirrhotic liver [68]. The majority of the dys-regulated miRNAs were down-regulated in HCC tissues, with the exception of miR-221 that was found to be up-regulated [68]. miR-122 was shown to be down-regulated in nearly 70% of the HCC specimens and all examined HCC-derived cell lines, implicating its role as tumor suppressor during the development of HCC [68].

In a comprehensive study, Pineau *et al.* performed miRNA expression in tissue samples (104 HCC, 90 adjacent cirrhotic livers, 21 normal livers) as well as in 35 HCC cell lines [69]. A set of 12 miRNAs (including miR-21, miR-221/222, miR-34a, miR-519a, miR-93, miR-96, and let-7c) was identified and were linked to disease progression from normal liver to cirrhosis to full blown liver cancer [69].

### Oncogenic and tumor suppressive miRNAs and their role in HCV-mediated hepatocellular carcinoma

Both oncogenic and tumor suppressive miRNAs play a significant role in HCV and HBV pathogenesis and liver carcinoma development, but limited studies have examined these aspects, along with the role of miRNAs in HCV infection-associated tumorigenesis and tumor suppression.

Varnholt *et al.* did quantitative analysis of miRNA expression profiles from formalin-fixed paraffin-embedded (FFPE) archival samples of HCV infected human primary liver tumors, consisting of premalignant dysplastic liver nodules and hepatocellular carcinomas [65]. 80 miRNAs were examined in a subset of tumors, which yielded 10 up-regulated and 19 down-regulated miRNAs compared to normal liver [65]. Five miRNAs (miR-122, miR-100, miR-10a, miR-198, and miR-145) were further examined in an extended tumor sample set of 43 hepatocellular carcinomas and 9 dysplastic nodules after selection [65]. miR-122, miR-100, and miR-10a were overexpressed whereas miR-198 and miR-145 were up to 5-fold down-regulated in hepatic tumors compared to normal liver parenchyma [65].

Expression levels of 188 miRNAs in liver tissues were obtained from 12 patients with hepatitis B virus (HBV)-related hepatocellular carcinoma (HCC) and 14 patients with HCV -related HCC with the help of real time PCR [70] combined with mRNA revealed two types of miRNA, one associated with HBV and HCV infections (n = 19), the other with the stage of liver disease (n = 31) [70]. On doing the pathway analysis of target genes for 13 down-regulated miRNAs in HCV infected patients the involvement of immune response, antigen presentation, cell cycle, proteasome, and lipid metabolism pathways [70], whereas the 6 down-regulated miRNAs in the HBV group regulated pathways related to cell death, DNA damage and recombination, and transcription signals [70].

Peng *et al.* also simultaneously profiled the expression of cellular miRNAs and mRNAs across 30 HCV positive or negative human liver biopsy samples using microarray technology [71]. They performed computational target predictions in order to construct a miRNA: mRNA regulatory network [71], in which the DE miRNA predominantly targeted the genes involved in chemokine (16 genes such as CXCL12 (C-X-C motif ligand 12 etc.), B cell receptor, Phosphatase and tensin homolog (PTEN) (13 genes), Interleukin (IL)-6, ERK (Extracellular signal related kinase)/MAPK (Mitogen activated protein kinase) (18 genes; Ras, Erk3 and STAT3 (Signal transducer and activator of transcription 3) etc.) and JAK (Janus kinase)/STAT signaling pathways, implying their important possible role in viral replication, propagation and latency of viral latency [71].

### Oncogenic miRNAs

#### Role of oncomir-miR-21 in cancer

miR- 21 is one of the first miRNAs detected abundantly in certain human cancers [72-75], and has been widely studied as an oncogenic miRNA and in relation to HCV mono-infection. Overexpression of miR-21 in variety of cancers [76-79] and tumors along with its role in increasing cell proliferation, migration and suppressing apoptosis in a cancer xenograft model suggests its vital role in different types of cancers, defining its role as an oncomir [80-84].

**Figure 3:**
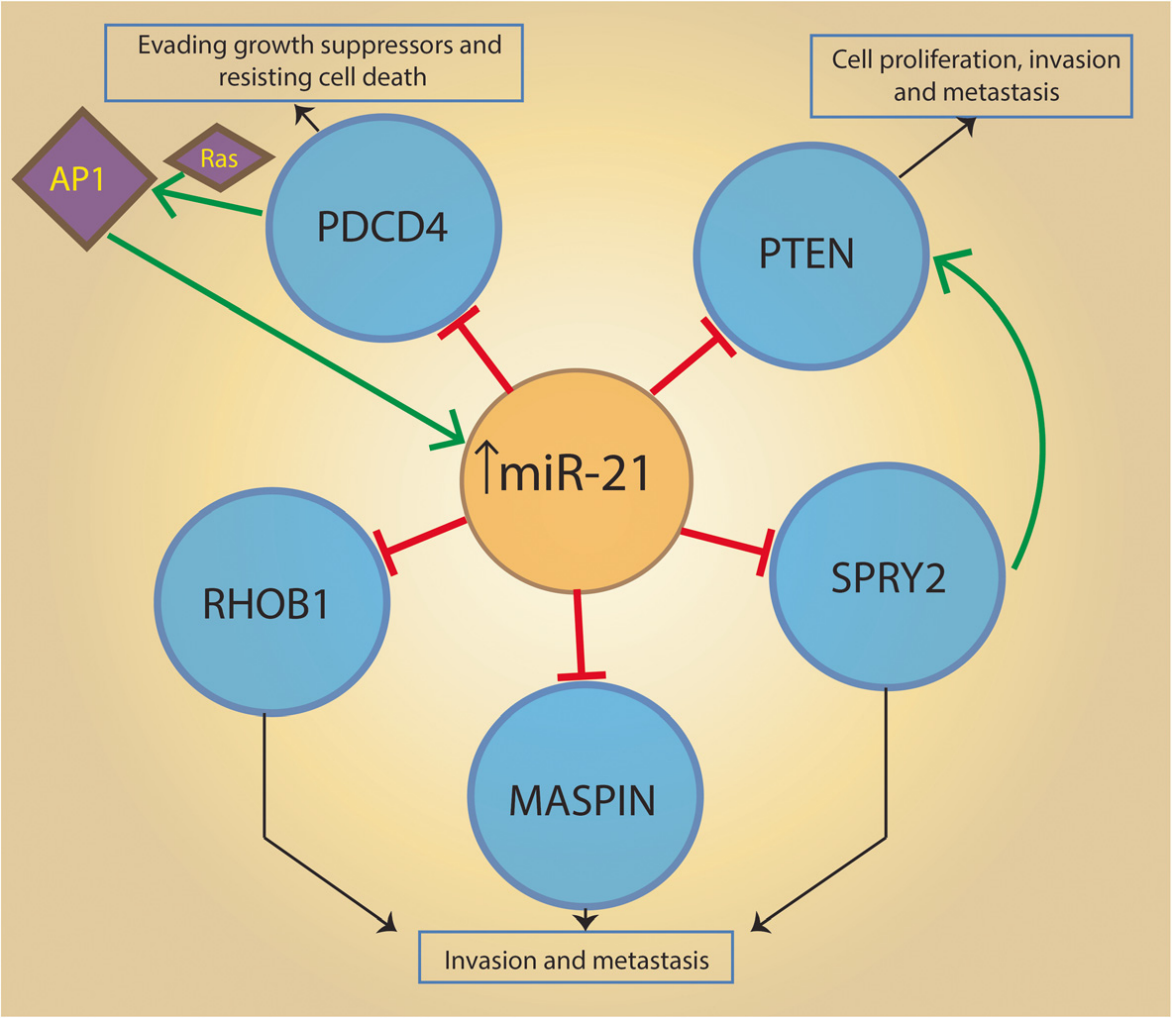
**Increased expression of miR-21 and its validated targets.** miR-21 expression can be activated by transcription factor AP-1, while miR-21 has negative downstream effects on the targets resulting in different biological outcome. The targets are represented as blue circles, while diamond represents the protein/transcription factor. The red bars represent down-regulation and the green arrows indicate the up-regulation.

miR-21 has also been noted to be highly overexpressed in HCC tumors and cell lines leading to increased cell proliferation and migration [85-87]. Meng *et al*. analysed miRNA expression profile in HCC tumor tissue versus normal liver and showed a greater than 5-fold increased miR-21 expression in the tumor compared to normal tissue samples [85]. Inhibition of miR-21 in cultured HCC cells was shown to increase the expression of the PTEN tumor suppressor, and decreased tumor cell proliferation, migration, and invasion [85]. Figure 3 shows the involvement of miR-21 and its notable validated targets in various signaling pathways related to cancer. PTEN was shown to be a direct target of miR-21, and to contribute to miR-21 effects on cell invasion [85] (Figure 3). Another validated target of miR-21 is Sprouty2 (SPRY2), which has also been shown to up-regulate PTEN [88,89] (Figure 3). Another target of interest tumor suppressor gene Ras Homolog gene family, member B (RHOB) was validated by Connolly *et al.* who demonstrated that down-regulation of miR-21 was associated with an up-regulation of RHOB in Huh-7 and HepG2 cell lines [90] (Figure 3). RHOB overexpression has been shown to inhibit tumor formation and reduction in migration, invasion and cell elongation [91]. miR-21 has also been shown to down-regulate the Programmed cell death protein 4 **(**PDCD4) tumor suppressor and its overexpression can cause stimulation of invasion and metastasis in colorectal cancer [80] (Figure 3). PDCD4 has also been shown to be up- regulated in apoptosis and cellular senescence, in addition to its tumor suppressive properties [92,93]. Another validated target for miR-21 is Maspin [94], implicated in cell invasion and metastasis (Figure 3). AP-1 (activator protein 1) is known to be an important regulator of cell proliferation, apoptosis and invasion [95,96] and has potential binding sites in precursor of miR-21, pri-miR-21 (Figure 3). These sites in pri-miR-21 suggest that miR-21 is a possible transcriptional target of AP-1. It also been shown to activate miR-21 transcription [97] (Figure 3).

#### Role of miR-221 in HCC

miR-221 is up regulated in human hepatocellular carcinoma (HCC) and proven target is cyclin-dependent kinase inhibitor CDKN1B/p27, whose down-regulation affects HCC prognosis [98]. Fornari *et al.* proved that the cyclin-dependent kinase inhibitor (CDKI) CDKN1C/p57 is also a direct target of miR-221 [99]. Up-regulation of miR-221 and miR-21 was shown to promote cell cycle progression, reduce cell death, and favour angiogenesis and invasion [100]. miR-221 has been demonstrated to function as an anti-apoptotic miRNA and its silencing can lead to increased apoptotic cell death. With the help of luciferase reporter assay and western blot analysis, pro-apoptotic protein Bcl-2-modifying factor **(**BMF) was identified as a direct functional target for miR-221 [101]. Inhibition of miR-221 led to an increase in expression of BMF and caspase 3 cleavage and *vice versa*. It was concluded that BMF plays a pro-apoptotic role by modulating the susceptibility of HCC cells to apoptotic stimuli through a caspase 3 dependent pathway.

#### miR-222 and oncogenesis

Microtranscriptome profiles of miRNA expression in HCC, adjacent cirrhotic tissue, and normal liver showed consistently high expression levels of miR-221 and miR-222 in tumor suggesting that both miRNAs can serve as oncogenic miRNAs [69]. To investigate the role of miR-222 in HCC, Wong *et al.* performed a detailed analysis on 99 primary HCC tumors and 94 tumor adjacent cirrhotic livers [102]. A 40-miRNA signature was identified that could discriminate tumors from adjacent cirrhotic liver tissue, and a step wise increase in miR-222 overexpression from cirrhotic livers to early HCC corroborated common miR-222 overexpression [102]. Increased miR-222 expression was also shown to correlate significantly with advanced stage HCC and with the shorter disease-free survival of patients [102]. Inhibition of miR-222 in Hep3B and HKCI-9 cell lines significantly retarded cell motility suggesting that miR-222 is a metastatic related miRNA [102]. Further investigations suggested that AKT (also known as Protein kinase B) signaling was the major pathway influenced by miR-222 [102]. The AKT protein PPP2R2A (Protein-coding, protein phosphatase 2A subunit B) was identified as a direct functional target for miR-222 [102]. It was suggested that the pro-metastatic effect of miR-222 is due to the ability to activate AKT signaling in HCC.

#### miR-17/92 and tumorigenesis

miR-17/92 cluster, which is composed of miR- 17-5p, miR-17-3p, miR-18a, miR-19a, miR-20a, miR-19b, and miR-92-1. miR-17-5p, was reported to be overexpressed in HCC. It has been noted in various tumors [78,103,104], and this overexpression has been shown to enhance the migration and proliferation, in addition to its involvement in both human and woodchuck hepatocellular carcinoma cell line [105]. Interestingly, the silencing of this miRNA cluster *in vitro* resulted in a 50% reduction in the proliferation and growth of hepatocytes [105]. Whereas, the specific inhibition of miR-17-5p resulted in decrease of the proliferation of HCC cells, supporting its role as an oncogenic miRNA [106]. miR-17-5p was shown to significantly activate the p38 mitogen-activated protein kinase (MAPK) pathway and increases the phosphorylation of heat shock protein 27 (HSP27) [106].

#### miR-30d in intra-hepatic metastasis

miR-30d was shown to be up-regulated in HCC and its expression was show to be highly associated with the intra-hepatic metastasis of HCC [107]. Galphai2 (GNAI2) was identified as a direct functional target of miR-30d [107]. Restoring the expression of GNAI2 in miR-30d-overexpressing cells inhibited the miR-30d mediated HCC cell growth and migration [107].

#### miR-192/miR-215 and miR-491 and enhancement of HCV replication

miR-192/miR-215 and miR-491 were shown to be capable of enhancing HCV replication in replicon cells [108]. miR-491 specifically suppressed the phosphoinositol-3 (PI3) kinase/Akt pathway [108]. The suppressive effect of miR-491 on HCV replication was abolished upon inhibition of PI3 kinase by LY294002 indicating that suppression of HCV replication by miR-491 was dependent on the PI3 kinase/Akt pathway [108].

#### Tumor suppressive miRNAs in Human HCC

It is now known that overexpressed miRNAs in cancers may function as oncogenes promoting cancer development by negatively regulating tumor suppressor genes and/or genes that guide cell differentiation or apoptosis. In contrast, the under-expressed miRNAs in cancers function as tumor suppressors and may inhibit cancers by regulating oncogenes and/or genes that guide cell differentiation or apoptosis. Several miRNAs have been described to be tumor suppressors.

#### miR-122

miR-122 has been demonstrated to be down-regulated in primary HCC tissues and in most hepatoma cell lines, suggesting its role as a tumor-suppressive miRNA in liver carcinoma [109-111]. miR-122 appears to play an important role in the regulation of metastatic characteristics such as cell migration and invasion as revealed by *in vitro* experiments on the HCC cell lines [109,112]. miR-122 has been shown to target a known promoter of metastasis, A Disintegrin and Metalloprotease 17 (ADAM17) and known to inhibit both tumor angiogenesis and cancer cell migration/invasion [113].

#### miR-29

**Figure 4:**
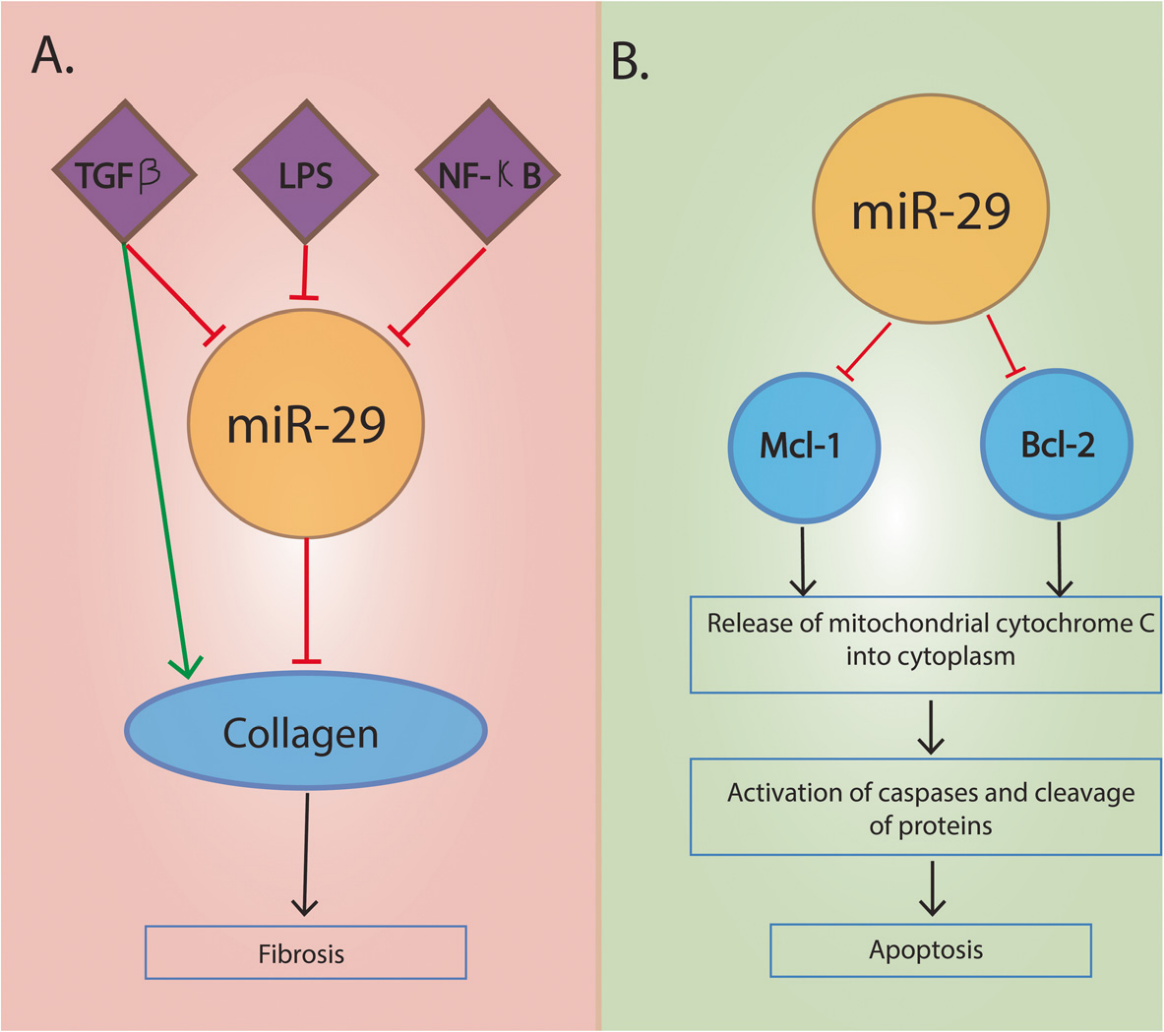
**Role of miR-29. A**. TGF-β, LPS and NF-κB stimulation leads to decreased miR-29 levels. Decreased miR-29 expression level is associated with increased collagen production leading to fibrosis. **B**. Increased expression of miR-29 is associated with down-regulation of two anti-apoptotic molecules, Bcl-2 and Mcl-1. Cytochrome c is released in the cytoplasm with the activation of caspases, suggesting that miR-29 may promote apoptosis through a mitochondrial pathway that involves Mcl-1 and Bcl-2. The targets are represented as blue circles, while diamond represents the protein/transcription factor. The red bars represent down-regulation and the green arrows indicate the up-regulation.

Roderburg *et al.* performed a systematic analysis of miRNA regulation in a mouse model of carbon tetrachloride induced hepatic fibrogenesis [114]. The study demonstrated that miR-29 family (miR-29a, miR-29b and miR-29c) is down-regulated both in livers of CCl4-treated mice as well as in mice that underwent bile duct ligation [114]. This down-regulation of miR-29 in murine model correlated with lower expression from miR-29 in liver from patients with advanced liver fibrosis [114]. In murine hepatic stellate cells (HSCs), down-regulation of miR-29 was mediated by transforming growth factor beta (TGF-β), inflammatory signals lipopolysaccharide (LPS) and nuclear factor kappa B (NF-κB) [114] (Figure 4A). Furthermore, overexpression of miR-29b in murine HSC resulted in down-regulation of collagen expression indicating its role as a regulator of liver fibrosis [114].

miR-29 family members (miR-29a, miR-29b and miR-29c) have been shown to be significantly down-regulated in HCC tissues [115]. In their study, Xiong *et al.* demonstrated that down-regulation of miR-29 was associated with poor disease free survival of HCC patients [116]. Re-introduction of miR-29 was associated with suppressed tumorigenicity and increased sensitivity of the HCC cells to apoptotic stimuli [116]. In addition to the above findings, B-cell lymphoma2 **(**Bcl-2) and Myeloid cell leukemia sequence 1 (Mcl-1) were validated as direct targets for miR-29 and the mitochondrial pathway shown to be activated in miR-29–promoted apoptosis (Figure 4B). Bcl-2 and Mcl-1 are anti-apoptotic proteins functioning through mitochondrial pathways and are shown to be up-regulated in different forms of cancer [117-119]. Their overexpression is associated with poor prognosis, causing the resistance of cells to apoptosis, favoring tumor progression [120,121]. While miR-29 down-regulates both of these gene targets thus promoting apoptosis, overexpression of the proteins encoded by the gene targets was shown to attenuate the effect of miR-29 [116].

#### miR-101

miR-101 was shown to be significantly down-regulated in HCC tissues [115]. It was also shown that the forced expression of miR-101 suppresses colony formation *in vitro* and tumor formation *in vivo*[115]. Mcl-1 was identified as a bona fide target of miR-101 [115]. Down-regulation of miR-101 was associated with up-regulation of Mcl-1, which is an anti-apoptotic molecule [115]. miR-101 was also shown to repress the expression of v-Fos FBJ murine osteosarcoma viral oncogene homolog (FOS) oncogene, a key component of the activator protein-1 (AP-1) transcription factor [122].

#### miR-139

Wong *et al.* demonstrated miR-139 was found to be significantly down-regulated in HCC patients and the expression was reduced much further in metastatic HCC samples when compared to primary HCC samples [123]. The role of miR-139 was investigated further with the help of HCC cell lines and with implantation mouse model [123]. miR-139 overexpression reduced the cell migration and invasion *in vitro* and also reduced the severity of lung metastasis from liver tumors in mice [123]. It was also demonstrated in the study that miR-139 interacts with 3′ un-translated region of Rho-kinase 2 (ROCK2) and reduced its expression in HCC cells [123]. ROCK2 is an important metastatic gene found to be up-regulated in HCC samples [124], therefore miR-139 can exert its tumor suppressive function through inhibiting of ROCK2.

#### let-7 g

Lan *et al.* demonstrated in their study that let-7 g may act as a tumor suppressor miRNA [125]. When HepG2 cells were transfected with let-7 g mimic, the proliferation of HCC cells was inhibited via the down-regulation of c-Myc [125]. Both mRNA and protein levels were reduced after transfection suggesting that the down-regulation occurred at both transcriptional and post-transcriptional level [125]. This effect was reversed after the introduction of a let-7 g inhibitor [125]. Over-expression of let-7 g also caused the up-regulation of p16INK4A, suggesting that the effect may be mediated through its direct regulation of c-Myc in the c-Myc-Bmi-1-p16 regulatory circuit [126]. These findings imply that let-7 g might function as an inhibitor of HCC cell proliferation through direct repression of c-Myc, which may lead to re-expression of the tumor suppressor p16INK4A [125]. In another study, Ji *et al.* demonstrated collagen type I alpha2 (COL1A2) as a direct target of let-7 g and addition of COL1A2 counteracted the inhibitory effect of let-7 g on cell migration [127].

The list of oncogenic and tumor suppressive miRNAs de-regulated in hepatocellular carcinoma is summarized in Table 2.

**Table 2:** Deregulated miRNA in hepatocellular carcinoma

**List of oncogenic and tumor suppressive miRNAs deregulated in hepatocellular carcinoma**			
**Oncogenic miRNA**			
Name	Expression in HCC	Target	References
miR-21	Up	PTEN, SPRY2, PDCD4, RHOB, MASPIN	[80,85,88,90,94]
miR-221	Up	CDKN1B/p27,CDKN1C/p57,BMF	[98,99,101]
miR-222	Up	PPP2R2A	[102]
miR-17/92	Up	HSP-27	[106]
miR-30d	Up	GNAI2	[107]
**Tumor suppressive miRNA**			
Name	Expression in HCC	Target	References
miR-122	Down	ADAM17	[113]
miR-29	Down	BCL-2, MCL-1	[116]
miR-101	Down	MCL-1, FOS	[115]
miR-139	Down	ROCK2	[123]
let-7 g	Down	C-myc, p16INK4A, COL1A2	[125,127]

*Abbreviations*: *PTEN* phosphatase and tensin homolog, *SPRY2* sprouty 2, *PDCD4* programmed cell death protein 4, *RHOB* ras homolog gene family, member B, *CDKN1B* cyclin-dependent kinase inhibitor 1B, *CDKN1C* cyclin-dependent kinase inhibitor 1C, *BMF* BCL-2 modifying factor, *PPP2R2A* protein-coding, protein phosphatase 2A subunit B, *HSP-27* heat shock protein 27, *GNAI2* galphai2, *ADAM17* A disintegrin and metalloprotease 17, *BCL-2* B cell lymphoma 2, *MCL-1* myeloid cell leukemia sequence 1, *FOS* v-fos FBJ murine osteosarcoma viral oncogene homolog, *ROCK2* rho kinase 2, *COL1A2* collagen type I alpha2.

### miRNA as future therapeutic targets

#### Therapeutic targeting of miRNA in HCV infection

While current standard therapy of HCV is pegylated IFN- α in combination with Ribavirin [128], success of the therapy depends upon the viral load before and during therapy and the genotype of the virus. Despite therapy, half of the patients fail to completely eradicate the virus [129,130] and both interferon and ribavirin are expensive and can cause severe side effects, limiting their clinical utility [128,131,132]. Numerous clinical trials have been undertaken to test promising new therapies including viral enzyme inhibitors, monoclonal and polyclonal antibodies, antisense RNA and therapeutic vaccination [133,134].

The discovery of positive regulatory role of miR-122 in HCV replication has led to the basis of developing miRNA–targeted therapeutic strategies for HCV infection. Inhibition of miR-122 can be achieved by the use of antisense technology, which can act at multiple levels to affect miR-122, such as binding to the mature, pre or pri-miR-122. The conventional antisense oligonucleotides (ASO) are low in efficacy and often require additional modification to improve their biological activity. The earliest report of miRNA inhibition using 2′–O-methyl (OMe) ASO described the injection of DNA oligonucleotides of the same length and complementary to the target let-7 miRNA in Drosophila embryos [135]. ASO were used to inhibit miR-122 in mice and termed as ‘antagomiRs’ [136]. Antagomirs were shown to reduce HCV RNA up to 84% in two independent subgenomic models, respectively [137]. Another approach 2′–O-methoxyethyl phosphorothioate (2′- MOE – RNA) modification is the oldest, simplest method and has been shown to effectively inhibit miR-122 activity in the liver [138]. Locked nucleic acid (LNA) modification represents a more advanced approach and is a class of ASO in which the 2′-0-oxygen is linked to 4′- C atom by the methylene bridge to form a rigid bicycle, locked into a C3′ –endo (RNA) sugar conformation. This modification leads to the formation of a thermodynamically strongest duplex formation with the complementary RNA known. miR-122 silencing can be achieved by LNA/2′–O-methyl mixmer, peptide nucleic acids (PNA), PNA–peptide conjugates, or a chimeric 2′Fluoro/2′OMe modified ASO. 2′Fluoro/2′OMe modified ASO is shown to be highly efficacious and has eight- fold potency compared to 2′-MOE ASO and can be further developed as a potential anti-HCV therapeutic agent [139]. LNA modified phosphorothioate drug, Miravirsen works by inhibiting miR-122 required for HCV RNA accumulation and replication [140]. Miravirsen is the first drug targeted against any miRNA that has entered into the clinical trials-Phase 2a. It was safe, well tolerated and provided prolonged antiviral activity with no signs of viral resistance and adverse effects being mild and infrequent [140].

To put into perspective, a recent study by Sendi; has suggested that miR-122 can have a dual role in the molecular pathogenesis of viral hepatitis [141]. While miR-122 has been shown to be involved in increasing HCV RNA replication [34,40,41], it can also decrease HBV replication through an inhibitory effect of p53 on HBV transcription through blocking of the binding of transcription factors like hepatocyte nuclear factors (HNFs) to HBV enhancer [142]. p53 can act as a tumor suppressor by inhibiting HBV replication and directly targeting cyclin-G1 [142], as well as Wnt/β-catenin [143], and N-myc downstream-regulated gene (NDRG3) pathways [144]. Thus while silencing of miR-122 has suggested as a treatment for HCV infection, on the flip side, miR-122 may also be considered as novel therapeutic targets for HBV by decreasing HBV replication. Although the results of this study are interesting, more studies need to be done and the results combined to further understand the mechanisms of actions of miR-122 in both HBV and HCV induced HCC.

## Conclusions

miRNAs play a remarkable role in regulating gene expression in both normal cellular processes and in viral infection. Viruses have evolved mechanisms to manipulate and subvert the host gene machinery by modifying both the gene expression and its regulatory machinery including miRNAs. HCV, particularly, appears to be influenced by the host cellular miRNA machinery to benefit its own replicative cycle- a classic example being miR-122, which is highly enriched in liver cells where it has been shown to be involved in the pathophysiology of HCV infection. miR-122 has also been tested in therapeutic clinical trials to treat HCV-infected chimpanzees [145] or human (Santaris, Phase-II) [140]. This documents for the first time that the miR 122 antagonist (Miravirsen) induced a prolonged, dose dependent reduction in virus titres in HCV-infected patients, which will probably lead to the first miRNA based therapy licensed for use [140]. Other miRNAs of interest miR-196b, 199a-3p, miR-141 and the miR-29 family can be used as miRNA therapy in the future. The combination miRNA therapy could also be used in conjunction with existing strategies, and possibly increase the efficiency of treatment. Even in the absence of miRNA-based therapeutics, miRNAs may serve as useful biomarkers for diagnosis, prognosis and possibly in defining response to therapy for HCV infection and may therefore become an important consideration in the future management of HCV. As the miRNAs are also the novel regulators of numerous immunological and non-immunological functions vital in the host immune response, a clear understanding of the role of these small molecules in the antiviral immunity may open doors to the development of new generation of biomarkers and antiviral treatment strategies, in addition to elucidating the mechanisms underlying virus-host interaction.

## Competing interests

The authors declare that they have no competing interests.

## Authors’ contributions

PG and NKS discussed the idea and PG drafted the manuscript putting all the ideas and literature together and worked till the conclusion of the final draft. NKS and MJC, apart from providing considerable intellectual input for improving the manuscript, also assisted in refining and finalizing the ideas. PG was fully responsible for sharing and putting the ideas together by liaising with NKS and MJC. All three authors actively contributed to the fruition of this manuscript. All authors read and approved the final manuscript.
